# Labelling of mental illness in a paediatric emergency department and its implications for stigma reduction education

**DOI:** 10.1007/s40037-017-0333-5

**Published:** 2017-03-07

**Authors:** Javeed Sukhera, Kristina Miller, Alexandra Milne, Christina Scerbo, Rodrick Lim, Alicia Cooper, Chris Watling

**Affiliations:** 1 Department of Psychiatry, Schulich School of Medicine and Dentistry, Western University, London, Ontario Canada; 2 Centre for Education Research and Innovation, Schulich School of Medicine and Dentistry, Western University, London, Ontario Canada; 3 Children’s Hospital, London Health Sciences Centre, London, Ontario Canada; 4 Division of Emergency Medicine, Schulich School of Medicine and Dentistry, Western University, London, Ontario Canada

**Keywords:** Stigma, Education, Labelling, Equity

## Abstract

**Introduction:**

Stigmatizing attitudes and behaviours towards patients with mental illness have negative consequences on their health. Despite research regarding educational and social contact-based interventions to reduce stigma, there are limitations to the success of these interventions for individuals with deeply held stigmatizing beliefs. Our study sought to better understand the process of implicit mental illness stigma in the setting of a paediatric emergency department to inform the design of future educational interventions.

**Methods:**

We conducted a qualitative exploration of mental illness stigma with interviews including physician, nurse, service user, caregiver and administrative staff participants (*n* = 24). We utilized the implicit association test as a discussion prompt to explore stigma outside of conscious awareness. We conducted our study utilizing constructivist grounded theory methodology, including purposeful theoretical sampling and constant comparative analysis.

**Results:**

Our study found that the confluence of socio-cultural, cognitive and emotional forces results in labelling of patients with mental illness as time-consuming, unpredictable and/or unfixable. These labels lead to unintentional avoidance behaviours from staff which are perceived as prejudicial and discriminatory by patients and caregivers. Participants emphasized education as the most useful intervention to reduce stigma, suggesting that educational interventions should focus on patient-provider relationships to foster humanizing labels for individuals with mental illness and by promoting provider empathy and engagement.

**Discussion:**

Our results suggest that educational interventions that target negative attributions, consider socio-cultural contexts and facilitate positive emotions in healthcare providers may be useful. Our findings may inform further research and interventions to reduce stereotypes towards marginalized groups in healthcare settings.

## What this paper adds

In a healthcare setting, mental illness stigma results from labelling patients with mental illness as time-consuming, unpredictable or unfixable. Perceived lack of self-efficacy in healthcare providers further perpetuates stigmatizing attributions. Educational interventions to reduce stigma may be more effective if they focus on patient-provider relationships and foster humanizing labels while increasing empathy skills. Work with individual providers is not enough to reduce stigma without considering socio-cultural influences.

## Introduction

Despite increasing attention and levels of public acceptance about the underlying cause of mental illness, mental illness stigma (negative attitudes and behaviours towards individuals with mental illness) continue to adversely impact patients and families [1–4]. A recent review found that stigma decreases help-seeking behaviours among individuals with mental illness(es) [5]. Stigma has been associated with a low quality of life and well-being [6, 7], persistent stress [8], interference with recovery [9], lower treatment adherence [10], shortened life span [11] and suicide [12]. Stigma also adversely impacts family relationships and contributes to caregiver fatigue [13].

Explanatory frameworks for stigma vary, ranging from individual cognitive models to motivational and structural models.[14, 15]. In particular, the work of Scheff and Goffman in the 1960s proposed modified labelling theory, emphasizing that stigma is an individual process enacted through social interactions [16–20]. Further scholars have refined Goffman and Scheff’s original work, describing stigma as resulting from converging forces including labelling of difference, linking of labels to undesirable characteristics, placement of labelled persons in distinct categories to accomplish separation and, lastly, the experience of discrimination that leads to unequal outcomes [17, 18].

Global efforts to reduce mental health stigma have been implemented by governments in most Western countries including the European Union, Australia, the USA and Canada [16]. Current models for stigma reduction include education and social contact. Education plays a role in reducing stigma by increasing knowledge about mental health and fostering counter-stereotypes about individuals with mental illness [21]. The success of stigma reduction interventions is typically measured by psychometrically validated scales at the individual level and through conceptual domains such as behaviour, penetration, psychological perspective, and knowledge at organizational and population levels [21]. While the public believes that stigma-reduction efforts have produced decreased stigma, research suggests that stigmatization of mental illness has shown a corresponding increase, which is perpetuated by associations between mental illness and dangerousness [22].

Existing research on stigma reduction education reveals several constraints on the power of current approaches to effect meaningful change. Educational interventions that provide information to dispel stereotypes regarding mental illness are considerably less effective for individuals who exhibit greater prejudice [14]. In contrast, social contact-based interventions that promote interpersonal contact with patients with mental illness are more effective than education alone [23, 24]. Social contact-based interventions have limited effect unless the contact is in person [25], the interactions are rewarding and enriching [16], and the individuals encountering each other are of equal power [26, 27], and pursue rewarding activities [28] towards a common goal [29]. Creating authentic learning environments that equalize the power differential between patients and healthcare providers is challenging. In most healthcare settings, patients and providers are rarely matched to each other, and when patients suffering from mental illness present in emergency or acute-care settings, there can be conflicting agendas and goals.

The success and sustainability of educational and contact-based interventions in healthcare organizations are therefore limited. Contact with patients with mental illness can lead to both positive and negative experiences for healthcare providers. If their experience reinforces their negative stereotype that patients with mental illness are dangerous, they may potentially further stereotype all patients with mental illness. Additionally, many interventions fail because they occur in organizational or sociocultural contexts that do not adequately address socio-cultural or societal influences [21]. A recent review of stigma reduction interventions in healthcare students and professionals found that education and contact-based interventions are often difficult to sustain, which raises questions as to whether cost of implementation may outweigh benefits [30].

We sought to address these limitations by considering deeper levels of stigma that may exist outside of conscious awareness. Implicit attitudes towards mental illness are gaining increasing recognition in the literature [31–35]. Since explicit attitudes and behaviour are often poorly assessed [36, 37], implicit measures such as the implicit association test (IAT) may play a role in future stigma reduction efforts. The IAT is an online tool that serves to bring an individual’s implicit attitudes regarding mental illness into their conscious awareness. The test pairs concepts with groups utilizing reaction time as primary outcome. Shorter latencies indicate stronger implicit associations. For example, the IAT measures associations between ‘dangerousness’ and either ‘mental illness’ or ‘physical illness’ [38].

Given the constraints of existing educational and contact-based interventions, our study sought to explore the social process of stigma including how stigmatizing attitudes and behaviours influence the healthcare experience. By exploring implicit stigma in a specific and targeted setting (paediatric emergency department) with diverse participant groups, we endeavoured to develop a deeper understanding of mental illness stigma to inform future educational interventions and reduce the adverse impact of stigma on patient outcomes.

## Methods

We utilized a constructivist grounded theory approach to explore the process of stigmatizing attitudes and behaviours in an emergency setting. Constructivist grounded theory is well suited to explore a social process that is not currently explained by a well-established theoretical construct [39].

Patients and families who utilize emergency department mental health services often report that stigma adversely impacts their experience [40, 41]. We chose the paediatric emergency department of an accredited Canadian academic health science centre to understand the process of stigma in a discrete environment that was representative of the gateway to acute-level healthcare. The principal investigator (JS) also maintains insider status as a child and adolescent psychiatrist and the research question emanated from observations during clinical activity regarding differential treatment of patients with mental illness and physical illness in this specific environment. A paediatric setting provided an opportunity to study the complex interplay between patients, caregivers and providers and was an ideal setting due to insider status, size, and scope of the clinical environment. Approval was obtained from the Western University Research Ethics Board (105881) to conduct the study.

Consistent with grounded theory principles, we used a purposeful, theoretical sampling approach. Our initial recruitment and interviews focused on patients and caregiver participants, addressing critiques of stigma research being uninformed by the lived experiences of patients and caregivers [42, 43]. We then invited multiple stakeholders including administrative staff, nurses and physicians through recruitment notices posted internally and through electronic means. Participants were asked to participate in 60-minute semi-structured interviews to explore stigma in a hospital setting. Analysis of our early interviews with patients and caregivers revealed the importance of clerical registration staff as part of the experience of stigma in a hospital setting. Therefore, we expanded our sample to include emergency department registration clerks.

Since our study was designed to move beyond existing stigma reduction paradigms and consider implicit attitudes about mental illness, we utilized a novel qualitative methodology that incorporated the mental illness IAT as an interview prompt. The use of specific triggers is a commonly used technique in grounded theory methodology to facilitate deeper discussion. Each interview began by reviewing a letter of information describing the nature of the IAT and that participation was voluntary. To promote safe disclosure, participants were advised that their IAT results would remain confidential and interviewers were prepared to debrief any potential emotional reactions regarding IAT results. In all interviews, participants were asked about the experience of taking the IAT and whether they felt that implicit associations played a role in mental illness stigma. In this study, the IAT was used as a prompt to trigger a richer interview about the process and experience of stigma and IAT results were not a part of the analysis. A separate study focused on participant responses to the IAT.

The team was composed of the principal investigator (JS), a child and adolescent psychiatrist, faculty member and PhD candidate in health professions education, as well as a paediatric emergency clinical leader (RL), research staff (KM), a clinical social worker (AC), and two registered nurse educators (AM and CS). Co-investigators also included CW who has expertise in health professions education research and qualitative research methods. Team members JS, KM, AM and CS conducted 24 individual semi-structured interviews from June to December 2015. To minimize power differential, a physician (JS) conducted the physician interviews, research staff (KM) conducted patient/caregiver interviews and nursing staff (AM and CS) conducted nursing and administrative staff interviews. Interviews followed a discussion guide developed from the initial literature review which included broad and open-ended questions exploring definitions of stigma, effects and possible interventions. The discussion guide was adapted iteratively as the study proceeded in accordance with a constructivist grounded theory approach [39].

Initial analysis was conducted by a team consisting of JS, KM, AM, and CS, beginning with line-by-line coding and utilizing constant comparative analysis to develop focused codes and working towards major themes. Key themes were shared with the entire team as analysis shifted to axial coding and the development of an explanatory theory that accounted for possible relationships between themes. Data collection continued until authors felt that sufficient data had been collected to enable a coherent and logical conceptual understanding of the process under study [44]. Findings were shared with a representative sample of participants via email to achieve triangulation. Participants from each group described general agreement with the synthesis of the results.

A total of 24 interviews were conducted across the various stakeholder groups. Four interviews were with service users (SU), 6 with caregivers (CG), 2 with clerical staff (CS), 2 with administrative staff (AS), 5 with physicians (MD) and 5 with nursing staff (RN). Participants were assigned a number based on the stakeholder group to which they belonged.

## Results

Broadly, most participants defined stigma as negative attitudes towards patients with mental illness and the behaviours that result from those attitudes. When asked to describe a situation that included stigma, participants described situations that fit with general definitions without contextual nuances specific to the emergency department. We found that key sociocultural and organizational influences on stigma – including media, history and a lack of mental health resources – contributed to fear and helplessness in providers. Patients with mental illness were labelled as time-consuming, dangerous, unpredictable and unfixable which led to avoidance behaviours. A perceived lack of self-efficacy in providers perpetuated frustration and helplessness, and further contributed to stigmatizing labels. Participants endorsed education as a key element of stigma reduction and focused on interventions at the patient-provider level.

### Sociocultural and organizational influences on stigma

Stigma was a consequence of complex forces that interact with each other at the sociocultural, organizational and individual levels. On a systemic level, participants described the impact of media and history as well as factors unique to mental health services including a lack of adequate funding and fragmentation of existing services. Notably, both patients and providers mentioned a lack of resources for mental health as contributing to stigma. Providers were more likely than other participants to draw a direct connection between lack of resources in the community and patients presenting to the emergency department seeking care which they did not feel equipped to provide.

*In healthcare field, and particularly working in emergency you like to help people. For the most part, there is a deficiency or a lack of any useful resources to help people. Emergency people don’t like dealing with entities they can’t help. (MD2)*

All participants acknowledged that stigma is worse in emergency settings. They recognized the emergency department as a physically and psychologically stigmatizing environment. The experience of seeking help and support, yet waiting for several hours without provider contact, was described as increasing frustration and worsening stigma.

*… We really do them an injustice in the emergency department because they get absolutely no treatment, they just sit there and wait and wait for hours and hours for psychiatry to come see them only to wait hours for a bed. (RN5)*

Even more broadly across every group of participants, fear was a predominant influence on the process of stigma. Individuals suffering with mental illness were afraid to disclose their illness and were prepared to be stereotyped. Similarly, staff mentioned their own fear and how it impacted their practice. One nurse described

*… an overriding fear … that somehow someone with a mental illness is going to negatively affect our lives. (RN4).*

Participants suggested that stigma in an emergency context was perpetuated by compassion fatigue. Emergency physicians and nurses described facing a high clinical burden in their clinical setting, leading to fatigue and burnout. The impact was a sense of frustration and helplessness that sometimes resulted in reluctance to engage with individuals with mental illness, as one physician noted:

*There’s a lot of emotional currency and cost in the interaction that may colour our willingness to go in. I think it can delay care and lead to negative talking about the patient even without having seen the patient. (MD3)*

### Stigma at the individual level

Although participant groups agreed on many themes, there was one striking difference: what physicians and nurses described as *labelling*, patients and caregivers perceived as *judgment*. Judgment was perceived to be a negative process that could internalize self-stigma including blame.

*Stigma is when somebody is judged for what they might look like or the way they act without the person understanding. It’s ignorance as far as I’m concerned. It’s not taking the time to sit back and say, you know, there is something wrong with that person (CG6)*

Many participants, particularly physicians and nurses, reflected that they associated patients with mental illnesses in the emergency setting with being unfixable. Additionally, some participants – particularly physicians – associated patients with mental illness as time-consuming, which was especially challenging in the efficiency-driven emergency department environment. The frustration caused by these labels appeared to increase stigma.

*A broken arm – I can fix it … With mental illness, there’s no sense of satisfaction whatsoever, because you emerge feeling despondent and like you’re despairing the family because we have nothing good to offer them. (MD4)*

### Reducing stigma through educational interventions

Participants agreed that a lack of education through health professions training contributed to stigma and shared that educating providers could reduce stigma by emphasizing humanism and teaching core empathy and communication skills. Several participants suggested that educational interventions that humanized mental health patients were more likely to succeed. They recognized that stigma was both a result of dehumanization and a contributor, and proposed that interventions fostering open dialogue and increasing empathy and compassion were important. Healthcare providers stressed the need to ‘treat every patient with respect’ and to avoid being ‘clouded by past negative experiences’ (MD3). And caregivers and service users reminded us that individuals with mental health problems ‘are humans too’ (SU5), and commented that physicians and nurses should approach them with compassion, and should ask ‘how can I communicate with this person more effectively in a kind and quiet manner?’ (CG2).

Participants identified a number of features they felt would enhance educational interventions: intervening early, increasing awareness of mental health presentations, promoting understanding of behaviours that result from mental illness, and being inclusive of all types of providers and staff. While participants recognized the impact of system issues such as lack of resources, inadequate funding, and fragmentation of services on stigma, they tended to perceive these factors as outside their sphere of influence; thus, their suggested educational interventions tended not to address these contextual forces. Instead, they focused on the patient-provider relationship, perhaps because they felt that was an area where they had the power to effect useful change.

## Discussion

**Fig. 1:**
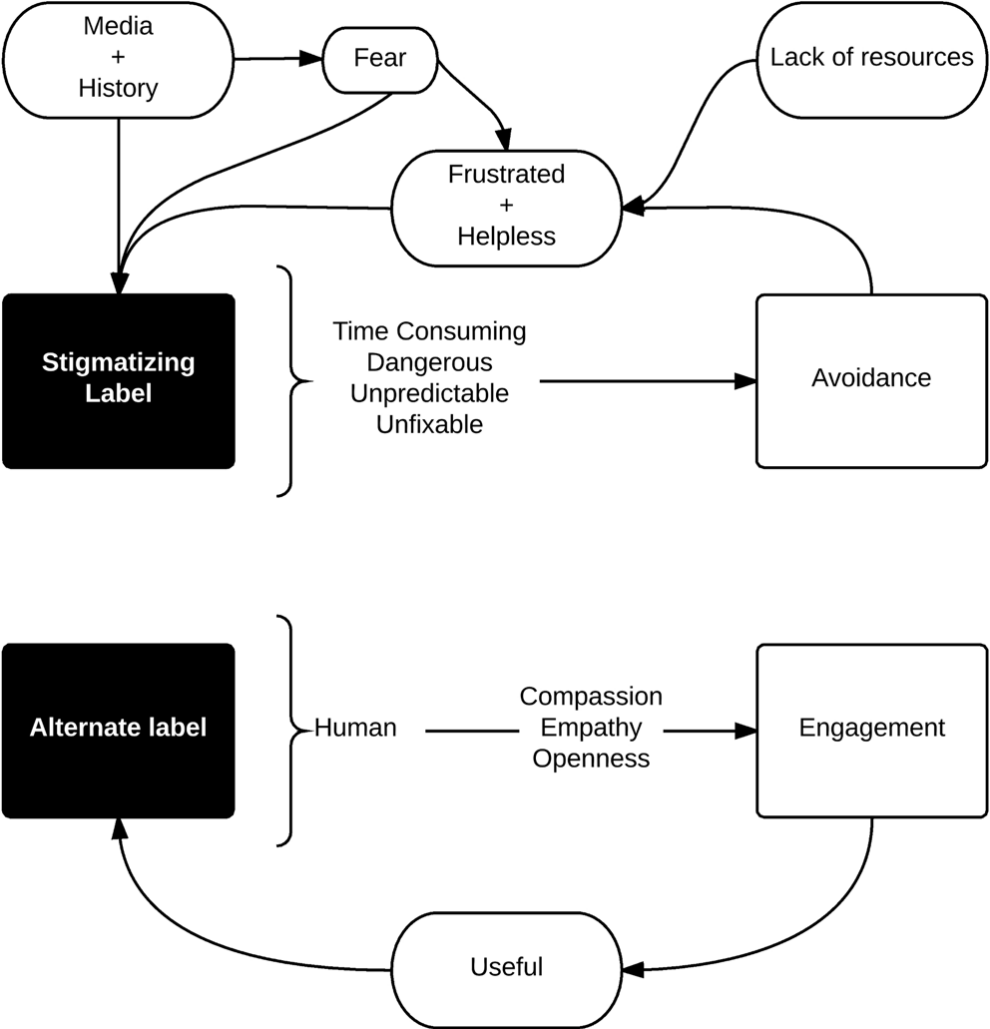
The confluence of socio-cultural forces and fear, frustration and helplessness lead to stigmatizing labels being attached to patients with mental illness and further avoidance behaviours which perpetuate frustration and helplessness. Possible solutions include re-framing patient-provider interactions towards humanizing labels, emphasizing compassion, empathy and openness. Fostering engagement may enhance provider self-efficacy

Our findings suggest that stigma in a paediatric emergency department results from a dynamic interaction between individual and sociocultural factors. A lack of system resources contributes to presentations of what are often complex, chronic problems to an acute-care, emergency setting that is perceived as unhelpful. Health providers experience frustration with recurring presentations, labelling patients with mental illness as time-consuming, unpredictable or unfixable. These labels result in avoidance behaviours which perpetuate fear, frustration and helplessness. How can we mitigate the effects of stigma and break this cycle? Educational interventions that bolster provider empathy and awareness of mental health may foster more alternative, humanizing labels and promote engagement instead of avoidance. We caution, however, that education of individual providers is not enough to reduce stigma; system, organization, and culture also demand attention. The model derived from this work (Fig. 1) illustrates the process of stigma as well as possible interventions.

### Labelling theory revisited: social context and relational power dynamics

Modified labelling theory emphasizes how labelling difference perpetuates discrimination and adversely impacts equitable and compassionate care for individuals with mental illness [17, 18]. Our results suggest that providers’ emotions and poor self-efficacy may perpetuate stigmatizing labels, and worsen distancing behaviours. Given this insight, we speculate that educational interventions that correct attributions and promote compassionate engagement may reduce stigma.

A focus on the patient-provider relationship resonates with labelling theory, which suggests that stigma is the result of a social process that involves a differential power dynamic between patient and provider [45, 46]. For example, in an emergency department a patient with mental illness who perceives prejudice from a provider may act more defensively, resulting in strained social interactions that perpetuate the process of stigma [47]. Existing literature reflects that labelling patients as ‘difficult’ may promote the process of social control for nurses who seek the prevention or resolution of deviance [48]. An emerging model proposed by Major and O’Brien [49] suggests that stigma is relationship and context specific and resides within a specific social context, rather than within individuals. Our findings shed light on the stigma-related behaviours of providers in the unique social context of an emergency department, where frustration is associated with a mismatch between a culture that emphasizes rapid symptom alleviation and a patient population that requires an inherently different approach.

Understanding the relationship between labels and context fits well with explanatory models that emphasize how stigma may play a role in protecting threat to psychological self by rationalizing negative group-based attitudes and discrimination [50, 51]. Using this model, patients with mental illness may be perceived as a threat against a culture that emphasizes time-limited medical interventions with immediate resolution. Therefore, emergency providers may be threatened by a large volume of patients that challenge their self-efficacy [52]. Existing research on attitudes towards mental health patients in emergency department highlights this issue, describing an environment that is not conducive to good mental health care [53]. Set in the context of a clinical environment where there are intrinsic and extrinsic incentives based on rapid response time and tangible outcomes [54–57], our findings suggest that this process may perpetuate further avoidance, rejection, frustration and stigmatization.

## Educational interventions should enhance self-efficacy and focus on patient-provider relationships

Existing research emphasizes that educational interventions that equalize power dynamics and address patients’ experiences of discrimination and prejudice may reduce stigma [58, 59]. Our findings add that provider self-efficacy may also be a useful target of educational interventions; deliberate efforts at perspective-taking and engagement, for example, may enable providers to more effectively mitigate the power differential that feeds stigma. Educational interventions that emphasize self-awareness and social perspective-taking may also limit unintentional avoidance behaviours. In short, interventions rooted in empathy may counter stigma. That healthcare providers, caregivers, and service users perceive that the relational aspects of care fall within their influence suggests that meaningful change in this realm is possible.

### Education is not enough to reduce stigma

Ultimately, any approach to reduce stigma must be multi-faceted and multi-level [18]. Our findings emphasize that educational interventions targeted at the individual level without considering the culture in which they are implemented are destined to fail. Any educational interventions in a healthcare environment should also consider the extrinsic incentives that reward briefer provider-patient interactions and perpetuate stigmatizing labels. Our results underscore the importance of realigning incentives, rewards and extrinsic motivators to promote the success of interventions that are designed to facilitate behavioural change.

Finally, our results draw attention to the continuing gap in training healthcare providers to be adept in managing and improving health systems. Despite our participants’ recognition of the social and organizational dimensions of stigma, they expressed an unsettling sense of helplessness to effect system-level change.

### Strengths and imitations

This study was designed as an exploratory study and sampled diverse groups of participants. Our limitations included the difficulty of capturing stigmatizing experiences for culturally and linguistically complex patients and families, as well as patients with communication difficulties. To address seasonal variation in paediatric mental health presentations, we attempted to sample across a prolonged time-span; however, our staff interviews took place in the fall-winter which includes the busier months of the year. While we attempted to address reflexivity by constructing a diverse team, our research team did not include patients and caregivers and therefore another potential limitation is the lack of patient/caregiver input into study design and results.
